# Transcranial Magnetic Stimulation in Bipolar II Disorder Treatment: A Case Report

**DOI:** 10.7759/cureus.45918

**Published:** 2023-09-25

**Authors:** Nga N Tran, Sydney Hutto, Landon R Thompson, Aaron Hawkins

**Affiliations:** 1 Medicine, Edward Via College of Osteopathic Medicine, Auburn, USA; 2 Family Medicine, Edward Via College of Osteopathic Medicine, Auburn, USA; 3 Psychiatry, Edward Via College of Osteopathic Medicine, Auburn, USA

**Keywords:** bipolar ii disorder, antipsychotic, bipolar, depression, theta burst, transcranial magnetic stimulation

## Abstract

The objective of this case report is to describe and document the use of transcranial magnetic stimulation (TMS) to aid in the treatment of bipolar II disorder. A 35-year-old male with a past medical history of attention-deficit/hyperactivity disorder (ADHD), post-traumatic stress disorder (PTSD), severe depression, and bipolar II disorder was presented to an outpatient psychiatric clinic 1.5 years after his initial TMS treatment for TMS maintenance therapy. He reported feeling depressed, brain fogginess, loss of concentration, fatigue, and constant changes in moods. He had tried multiple antidepressants and antipsychotics, seen several therapists, and underwent electroconvulsive therapy in 2014 with no improvement. In August 2021, he underwent the standard TMS protocol with 36 treatments and noticed significant improvement in his symptoms. He followed up with his psychiatrist who placed him on quetiapine 400 mg, lurasidone 120 mg, topiramate 100 mg, Adderall 20 mg, Wellbutrin 150 mg, propranolol 20 mg, and Klonopin 0.5 mg for management. However, after starting these medications, he noticed a loss of concentration, not being able to think straight, fatigue, depression, and a change in moods. In January 2023, the patient underwent maintenance TMS treatment with theta bursts (TBS). The treatment protocol consisted of 10 sessions for 3 ½ minutes each, 20 trains, 10 bursts, and eight seconds between intervals. He completed his treatment and reported feeling great and like himself again. Two weeks following treatment, he reported that his brain fog had resolved, hypomanic episodes had lessened, and depressive moods had been occurring less often. Due to improvement, topiramate and lurasidone were discontinued and the patient will continue with monthly follow-ups to monitor his progress. TMS appears to be a promising treatment option for bipolar disorder.

## Introduction

Transcranial magnetic stimulation (TMS) is a non-invasive procedure to stimulate nerves in the brain used in both psychiatric treatment and brain mapping [1]. TMS employs a primary coil, which uses an electric current to generate a magnetic field that is transferred to neural tissue that acts as a secondary coil [2]. These electromagnetic bursts act upon neurons with curved axonal processes at a right angle to the primary coil [3]. The electrical field induced in the neural tissue depolarizes the axons, eventually resulting in an action potential if the threshold is reached [4]. The ability to reach 120% motor evoked potentials (MEP) from 80% MEP is dependent on the type of coil and intensity of electrical current used [5]. Repeated stimulation of neurons can lead to an increase in synaptic strength and an increase in the amplitude of excitatory postsynaptic potentials [2].

TMS can be employed in both a single pulse and repetitive TMS (rTMS) [1]. Physiologic response to rTMS is dependent on the stimulation frequency. Low-frequency stimulation has an inhibitory effect, whereas high-frequency stimulation (>5Hz) produces an excitatory effect [2]. rTMS utilized in the treatment of psychiatric disorders is most commonly directed at the left dorsolateral prefrontal cortex (DLPFC) [3]. TMS over the DLPFC may stimulate the cortico-striatal-thalamo-cortical loop, which plays a pivotal role in the regulation of emotions via dopamine [4]. A single pulse TMS can be used to determine the location of the DLPFC, and intensity needed to evoke an action potential by obtaining an MEP in the primary motor cortex [6]. The lowest TMS intensity to attain an MEP is representative of the motor threshold, which can be applied to the neurons of the DLPFC [2,5]. Theta burst stimulation (TBS) is a newer protocol used in the maintenance treatment of psychiatric disorders, producing TMS pulses that mimic the theta rhythm of the hippocampus [2]. TBS treatment is approximately three minutes compared to the standard 40-minute session in TMS treatments [7]. TBS produces a similar efficacy of treatment when compared to traditional TMS and may decrease the number of patients lost to follow-up [8]. The FDA has approved the use of TMS in the treatment of major unipolar depression and obsessive-compulsive disorder [1]. However, TMS is used as an off-label treatment modality in a plethora of other indications, including post-traumatic stress disorder, bipolar disorder, fibromyalgia, Parkinson’s disease, tinnitus, multiple sclerosis, schizophrenia, and more [8]. Though there is a significant effect seen in the treatment of mood disorders such as major depressive disorder, there is also a relapse rate of up to 30% [9]. Maintenance therapy is a current topic of research that may help reduce the rate of relapse [9].

Bipolar disorder I is diagnosed in the presence of at least one manic episode, defined by the Diagnostic and Statistical Manual of Mental Disorders, Fifth Edition, Text Revision (DSM-5-TR) as “a distinct period of abnormally and persistently elevated, expansive, or irritable mood and abnormally and persistently increased energy” spanning at least one week [10]. This is differentiated from bipolar disorder II, which is diagnosed based on the DSM-5-TR as “at least one hypomanic episode and at least one major depressive episode” in which the hypomanic episode is “a distinct period of abnormally and persistently elevated and expansive or irritable mood and abnormally increased activity or energy lasting for at least 4 consecutive days and present most of the day, nearly every day” [10]. The diagnosis of bipolar I or II is diagnosed via clinical history and a series of diagnostic questions addressing different symptoms of depression and mania [11]. Fluctuation of symptoms can cause antagonistic effects on the patient’s life and increase the risk of suicide by up to 14% and violent deaths as four times as likely [12]. Bipolar disorders normally present in patients between 12 and 30 years old and require chronic management, often with pharmacologic therapy [12].

Management of bipolar disorder can be focused on either the acute treatment of mood episodes, including mania, hypomania, and depression, or maintenance therapy to prevent episode recurrence [13]. Acute episodes are primarily treated with mood stabilizers or antipsychotic drugs either in combination or individually based on patient response and severity of the episode, and side effect profiles [13]. Effective acute treatments may be used as maintenance therapy to prevent future episodes, this commonly includes lithium, which has been shown to be effective in preventing manic, depressive, and mixed relapse as well as reducing suicide risk [14]. Patients who are managed well on medication are recommended to remain on this therapy indefinitely to prevent relapse [14]. Long-term lithium use can cause adverse effects, including but not limited to polyuria, nephropathy, tremor, extrapyramidal symptoms, lithium intoxication, hypothyroidism, goiter, weight gain, hypercalcemia, hyperparathyroidism, and electrocardiogram T wave inversion [15]. Patients undergoing lithium maintenance therapy are encouraged to test urea, electrolytes including calcium, creatinine, parathyroid hormone, and lithium levels every three to six months [16]. TMS treatment is utilized after attempting multiple other medications for management. Clinical trials of TMS for bipolar disorder treatment primarily suggest benefits of depressive symptomatology in bipolar II, with mixed results of manic symptoms [16].

## Case presentation

A 35-year-old male with a past medical history of attention-deficit/hyperactivity disorder (ADHD), post-traumatic stress disorder (PTSD), severe depression, and bipolar II disorder presented to an outpatient psychiatric clinic for TMS. He reported feeling depressed, brain fogginess, loss of concentration, fatigue, and constant changes in moods. He had tried multiple antidepressants and antipsychotics, seen several therapists, and underwent electroconvulsive therapy in 2014 with no improvement. The patient was involved in a severe car accident in 2014, which is when he noticed his symptoms were exacerbated. He expressed feeling anxious about being in cars and was having at least two nightmares a week about the event. In early 2021, he was admitted into an inpatient psychiatric center for a suicide attempt.

In August 2021, he underwent the standard TMS protocol with 36 treatments (see Table 1) and noticed significant improvement in his symptoms. He was having fewer depressive symptoms, improved cognitive fogginess, and noticed fewer hypomanic episodes. He followed up with his psychiatrist who placed him on quetiapine 400 mg, lurasidone 120 mg, topiramate 100 mg, Adderall 20 mg, Wellbutrin 150 mg, propranolol 20 mg, and Klonopin 0.5 mg for management. However, after starting these medications, he noticed a loss of concentration, not being able to think straight, fatigue, depression, and a change in moods. The patient wanted to try TMS again.

**Table 1:** Standard TMS treatment vs. maintenance TMS treatment TMS: transcranial magnetic stimulation.

Standard TMS treatment	Maintenance TMS treatment
36 sessions	10 sessions
40 minutes per session	3 ½ minutes per session
10 Hz	5 Hz
10 bursts per second for 4 seconds	10 bursts every 2 seconds
26 seconds intervals	8 seconds intervals
75 trains	20 trains
3,000 pulses per session	600 pulses per session

In January 2023, the patient returned to the clinic to begin his maintenance TMS treatment with theta bursts (TBS). The patient was educated on drop-off periods during traditional TMS treatments, which occurs when a patient can notice symptoms will begin to worsen before there is improvement but during TBS, there is a lack of drop-off periods. The treatment protocol consisted of 10 sessions for 3 ½ minutes each, 20 trains, 10 bursts, and eight seconds between intervals, which is different from the standard protocol (Table 1). He completed his treatment and reported feeling great and like his old self. The patient’s quetiapine was reduced to 300 mg, lurasidone was reduced to 80 mg, topiramate was reduced to 25 mg, and the rest of the medications remained the same.

Two weeks after the patient completed his first maintenance TMS treatment with theta bursts, he reported feeling better. His cognitive fog had resolved, hypomanic episodes had lessened, and depressive moods had been occurring less often. Due to improvement, topiramate and lurasidone were discontinued and the patient was directed to continue with monthly follow-ups to monitor his progress. While there is no current protocol on how to proceed with maintenance TMS treatments, it is currently recommended if symptoms return.

## Discussion

Traditional pharmacotherapy for bipolar disorder typically involves the use of mood stabilizers, antipsychotics, and antidepressants, which can be effective in controlling symptoms but may have significant side effects [15]. Mood stabilizers such as lithium can cause side effects such as tremors, weight gain, and kidney damage, while antipsychotics may cause side effects such as sedation, weight gain, and movement disorders [16]. Antidepressants can also cause side effects such as insomnia, sexual dysfunction, and suicidal thoughts [15]. Because it is minimally invasive, TMS has a low side effect profile. The side effects are headache, muscle twitching, and scalp discomfort. The side effects of TMS, however, are transient and resolved when the treatment session is over.

TMS is an underused and important therapy for patients with mental illnesses and disorders. TMS works by stimulating nerves in specific areas of the brain with a magnetic field [1]. This can lead to stronger synaptic connections and an improvement in symptomatology. TMS has been FDA-approved for the treatment of major unipolar depression and obsessive-compulsive disorder [2]. However, it is also used off-label for a plethora of other medical states, including bipolar disorder, PTSD, fibromyalgia, and more. TMS can be an encouraging option for patients who have either not received adequate symptom resolution or have experienced intolerable side effects from traditional pharmacotherapy.

One of the several advantages of TMS is that it specifically targets areas of the brain that are dysfunctionally associated with a given mental illness. It does so with high accuracy. For example, in the case of bipolar disorder, TMS is purposefully positioned to affect the left dorsolateral prefrontal cortex [4]. This region plays a role in the regulation of emotional control. Clinical evidence is building and suggests that TMS can be both efficacious and safe in the reduction of depressive symptomatology in bipolar II [16]. The ability to target specific areas of the brain with TMS makes it a promising option for the treatment of a range of mental illnesses and disorders [16].

## Conclusions

This case report discusses the use of TMS for the treatment of a 35-year-old male with a past medical history of ADHD, PTSD, severe depression, and bipolar II disorder. After undergoing a standard TMS protocol with 36 treatments, the patient experienced significant improvement in his symptoms. He later underwent maintenance TMS treatment with theta bursts and reported feeling like his old self. Topiramate and lurasidone were discontinued and the patient will continue with monthly follow-ups to monitor his progress. TMS appears to be a promising treatment option for bipolar disorder, primarily suggesting the benefit of depressive symptomatology in bipolar II, with mixed results of manic symptoms.
